# The Effects of Age, Period, and Cohort on the Mortality of Cervical Cancer in Three High-Income Countries: Canada, Korea, and Italy

**DOI:** 10.1155/2021/8829122

**Published:** 2021-01-04

**Authors:** Jinyao Wang, Zhiqiang Bai, Xudong Gao, Nianping Zhang, Zhenkun Wang

**Affiliations:** ^1^Public Health Teaching Center, Department of Medicine, Shanxi Datong University, Xingyun Road, Datong 037009, China; ^2^School of Life Sciences, Shanxi Datong University, Xingyun Road, Datong 037009, China; ^3^School of Health Science and Nursing, Wuhan Polytechnic University, Hubei Wuhan 430023, China; ^4^Department of Medicine, Shanxi Datong University, Xingyun Road, Datong 037009, China; ^5^Party Committee Organization Department, Tongji Hospital, Tongji Medical College, Huazhong University of Science and Technology, Wuhan 430030, China

## Abstract

**Background:**

As the second most common gynecologic cancer worldwide, cervical cancer has led to morbidity and mortality in thousands of women. Our study is aimed at comparing the long-term trends of mortality rates for cervical cancer in three high-income countries—Canada, Korea, and Italy—and analyzing the detached effects of chronological age, time period, and birth cohort by age-period-cohort (APC) analysis.

**Methods:**

Joinpoint regression was used in this study, and the age-period-cohort model combined with the intrinsic estimator method was also applied to estimate the detached effect of each age, time period, and birth cohort on cervical cancer mortality.

**Results:**

For the overall trends of ASMRs for cervical cancer, the rates for Canada and Italy generally decreased during the whole observation periods while the rate for Korea exhibited a significant increase from 1986 to 2003. The APC analysis suggested that the cancer mortality risks consistently increased with age in the age groups including women aged 20 to 50 years in all areas. The period effect exhibited a general upward trend for both Korea and Italy, while a decreased trend was observed for Canada during the whole observation period. The mortality risk generally decreased with birth cohort, except there was a slight increase for younger generations in the three countries.

**Conclusions:**

Our study shows that the overall decrease in the cohort effect may have contributed to the reduced mortality rate for Italy and Canada, and the increased period effects and cohort effect in younger generations may have led to the increase in cancer mortality rate for Korea.

## 1. Introduction

Cervical cancer is the fourth most common cancer, and it ranks as the second most incident gynecological cancer after only breast cancer in the female population worldwide [1–3]. It has been estimated that approximately 527,000 females were newly diagnosed with cervical cancer and more than 266,000 females died from cervical cancer in 2012 globally [4]. According to the International Agency for Research on global cancer estimates, there were an estimated 569,847 new cases of cervical and 311,365 deaths worldwide in 2018, accounting for 3.2% of new cancer cases and deaths in females [5]. More than 60,000 females are newly diagnosed with cervical cancer and over 25,000 women die from the disease every year in Europe, and cervical cancer is estimated to be one of the top 10 leading causes of cancer death in some Asian countries [6, 7]. While widespread cytology screening has dramatically reduced the morbidity and mortality of cervical cancer in many developed countries, it remains a significant health risk with relatively high burden of disease among European and Asian female populations, particularly affecting young women [8–10].

In our study, the joinpoint regression model was applied by performing several permutation tests to identify the possible number of joinpoints in which significant changes in the linear slope of the incidence and mortality trends were explored during the whole study period [11, 12]. Furthermore, the APC model was also applied to assess the detached effects of the three factors in cervical cancer mortality rates. The age-period-cohort (APC) model is a common statistical method which has been widely utilized in fields of demography and epidemiology of human populations [13]. To describe the temporal trends of cervical cancer mortality of high-income countries in East Asia and Western countries and to better compare these trends in different areas, we selected Canada, Korea, and Italy for this analysis. Furthermore, we established the age-period-cohort (APC) model combined with the intrinsic estimator algorithm in our study to separate the independent effects of chronological age, time period, and birth cohort and to investigate the longitudinal trends of cervical cancer mortality in three high-income countries from1986 to 2015.

## 2. Materials and Methods

### 2.1. Data Source

All research data on cervical cancer mortality used in this study were obtained from the WHO Mortality Database for Canada, Korea, and Italy. (https://www-dep.iarc.fr/ WHOdb/WHOdb.htm). Furthermore, to better compare and analyze the temporal trends of cervical cancer mortality in high-income Asian and Western countries, we selected Canada, Korea, and Italy as the three countries to analyze from 1986 to 2015 [2].

Cervical cancer was coded as C53 according to the 10^th^ revision of the International Classification of Diseases (ICD-10) [14]. In our study, cases in patients under 20 years old and above 80 years old were excluded because cervical cancer is very rare in patients under 20 years old and patients over 80 years old might die from many other competing causes [14, 15]. Thus, only cases in patients aged 20-79 years were included in our study. For the analysis of mortality trends with the APC model, all cases were stratified into 5-year age groups from 20 to 80 years old and 5-year calendar periods from 1986 to 2015 [1]. Furthermore, to increase the comparability of the three regions, age-standardized mortality rates (ASMRs) were calculated by Segi [16] and Doll et al. [17], which were adjusted to the world population structure.

### 2.2. Statistical Analysis

In our study, the joinpoint regression model was applied to identify the significant changes in mortality trends of cervical cancer. The annual percent change (APC) was also used to compare changes for cervical cancer mortality by age group within each time period. Furthermore, the Joinpoint Regression software ver. 4.2.0.1 (Surveillance Research Program, National Cancer Institute, Bethesda, MD) was used [18].

Moreover, the APC model was also applied to explore the changing patterns of mortality trends in China and Italy for cervical cancer. The APC model is an important epidemiological and statistical tool based on Poisson distribution, and it can reflect secular changing trends of diseases in age, period, and cohort under adjustment for age, period, and cohort [2]. Usually, the model can be expressed as

(1) $$\begin{array}{c} M_{ij}=\mu +\alpha_{i}+\beta_{j}+\gamma_{k}+\varepsilon_{ij}, \end{array}$$

where *M*_*ij*_ represents the mortality for the *i*th age group (*i* = 1, 2, 3, ⋯, 12) at the *j*th (*j* = 1, 2, ⋯, 12) period of observed data, *μ* represents the intercept or adjusted mean mortality, *α*_*i*_ is the age effect or the coefficient of the *i*th age group, *β*_*j*_ is the period effect or the coefficient of the *j*th period, *γk* (*k* = *a* − *i* + *j*; *k* = 1,2,3, ⋯, *a* + *p* − 1) is the cohort effect or the coefficient of the *k*th, and *ε*_*ij*_ is *a* random error with the expectation *E*(*ε*_*ij*_) = 0. A linear relationship exists between age, period, and cohort, that is,

(2) $$\begin{array}{c} \text{Period}=\text{Age}+\text{Cohort}. \end{array}$$

Thus, it is difficult to analyze the unique set for every age, period, and cohort effect. This is called the nonidentification problem, and it has been widely discussed and addressed in demography, statistics, and epidemiology [13]. Fu [19] proposed recent developments in the APC model in the derivation of a new APC estimator named the intrinsic estimator (IE); this is a new approach for the estimation of the APC model which is based on estimable functions and the singular value decomposition of matrices [20]. In our study, all statistical analyses were performed with the STATA 12.0 software (StataCorp, College Station, TX, USA). The values of the Akaike information criterion (AIC), Bayesian information criterion (BIC), and deviance were calculated simultaneously to evaluate the goodness of fit of the model.

## 3. Results

### 3.1. Cervical Cancer Mortality Rates in Three Areas

**Figure 1:**
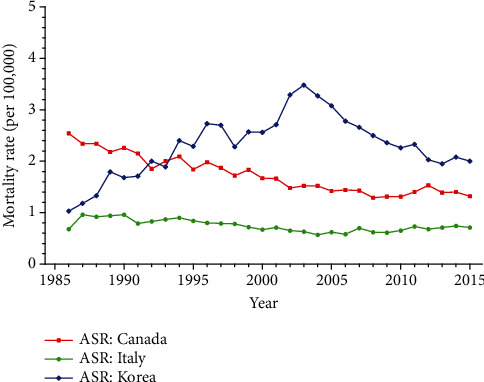
Trends of age-standardized mortality rates for cervical cancer per 100,000 female population in three high-income areas, 1986-2015.

The secular trends in age-standardized mortality rate (ASMR) for cervical cancer in women of all ages from 1986 to 2015 are depicted in Figure 1. Appendix Tables S1–S3 in the Supplementary Materials show the age-specific mortality rates for cervical cancer by year of death in three regions—Canada, Korea, and Italy. As is presented in Figure 1, the age-standardized mortality rates for cervical cancer in Canada and Italy generally decreased during the entire observation period, while Korea experienced a significant increase from 1986 to 2003. Furthermore, Korea had the highest mortality rate during the period from 1994 to 2015, whereas the ASMR observed in Italy was lower than that of the other two countries during the entire observation period.

### 3.2. Trends of Cervical Cancer Mortality Rates by Joinpoint Regression

To further explore the significant changes in the linear slope of mortality trends, the joinpoint regression model was applied and the results are presented as Table 1. As shown in Table 1, the ASMRs of cervical cancer for Canada among women of all ages decreased from 2.54 to 1.32 per 100,000 females during the whole observation period from 1986 to 2015 (AAPC, -1.9; *P* < 0.05; 95% CI, -2.4~-1.4). The ASMRs for Korea and Italy increased from 1.03 to 2.00 (AAPC, 2.2; *P* < 0.05; 95% CI, 0.9~3.5) and 0.68 to 0.71 (AAPC, 0.0; *P* > 0.05; 95% CI, -1.2~1.3) per 100,000 females, respectively.

In addition, the results by segmented analysis showed that the ASMRs for Canada significantly decreased during the periods from 1986 to 2008 (APC, -2.7; *P* < 0.05), whereas the mortality rates for Korea exhibited two significant increases during the periods of 1986-1989 (APC, 18.9; *P* < 0.05) and 1989-2003 (APC, 4.8; *P* < 0.05). Furthermore, the mortality changes for Italy demonstrated a significant increase during the periods from 2005 to 2015 (APC, 1.9; *P* < 0.05), followed by a significant decreasing trend from 1988 to 2005 (APC, -2.8; *P* < 0.05).

### 3.3. The Age, Period, and Cohort Effects on Cervical Cancer Mortality by the APC Model

**Figure 2:**
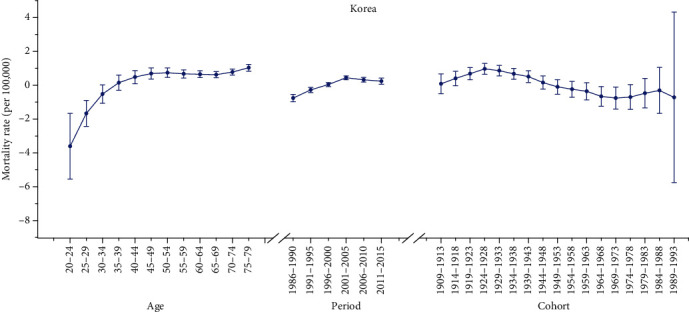
Age, period, and cohort effects on cervical cancer mortality in Korea (bars around the point estimate indicate the 95% confidence intervals).

**Figure 3:**
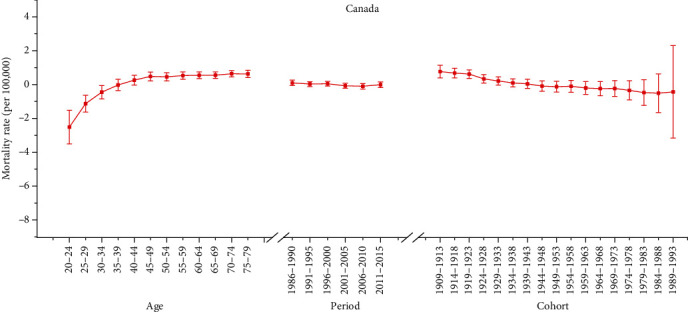
Age, period, and cohort effects on cervical cancer mortality in Canada (bars around the point estimate indicate the 95% confidence intervals).

**Figure 4:**
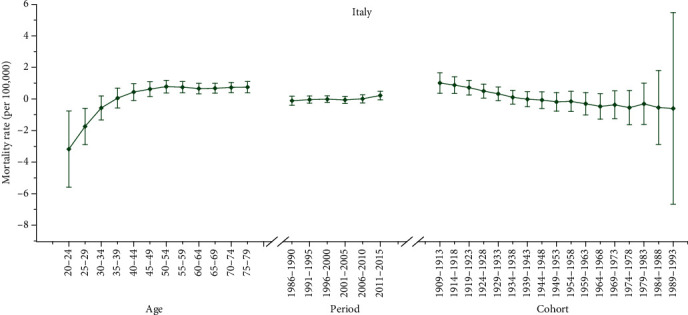
Age, period, and cohort effects on cervical cancer mortality in Italy (bars around the point estimate indicate the 95% confidence intervals).

Furthermore, we applied the APC model combined with the intrinsic estimator method to estimate the coefficients for age, period, and cohort effects. The results of APC analysis for age-specific mortality for cervical cancer in three countries are described in Table 2. Moreover, Figures 2–4 reflect the age, period, and cohort effects based on Table 2. The separate effect of each element was investigated as follows.

#### 3.3.1. Age Effect

As presented in Figures 2–4, the age effect increased consistently with age in the age groups including women aged 20 to 50 years in all areas. In Korea and Italy, the effect increased significantly before the age of 50 years and peaked in the age group of 50-54 years for Italy (seen in Figures 2 and 4). As presented in Table 2, the age effect in Korea generally rose in all age groups, but there was a slight decrease from 0.74 to 0.62 in the 50-54– to 65-69–year-old age groups. Moreover, as shown in Figure 3, the effect for Canada consistently increased in all age groups from 20 to 79 years, except there were two slight decreases from 45-49 to 50-54 and from 70-74 to 75-79 years old. Similar with Korea, the age effect in Italy generally rose in all age groups, except there was a slight decrease from 0.78 to 0.66 in the age group from 50-54 to 60-64 years old (seen in Table 2). Generally, the lowest coefficient of estimation for the age effect was observed in the group including women aged 20-24 years in all three areas.

#### 3.3.2. Period Effect

As shown in Table 2, the period effect exhibited a general upward trend for both Korea and Italy during the entire observation period while a slight decreasing trend was identified for both Korea (from 0.44 to 0.23) and Italy (from -0.01 to -0.06) during the periods from 2001-2015 to 1996-2005, respectively. Furthermore, the period effects in Canada declined during the whole study period from approximately 0.10 to -0.01, while two slight increasing trends were identified during the periods from 1991 to 2000 (from 0.03 to 0.05) and during the periods from 2006 to 2015 (from -0.10 to -0.01).

#### 3.3.3. Cohort Effect

As shown in Figures 2–4, the cohort effect suggested that the mortality risk generally decreased from 1909-1913 to 1989-1993 for most birth cohorts in the three areas. Furthermore, the mortality risk consistently declined in the cohort born before 1953 for both Italy and Canada (seen in Figures 3 and 4). A slight ascending trend was observed for those born from 1949-1953 to 1954-1958 and from 1964-1968 to 1969-1973. A small ascending trend was also observed in both Italy and Canada for more recent birth cohorts (seen in Figures 3 and 4). Overall, the mortality risk by birth cohort showed a declining trend except for some periods and the effects all peaked in the cohort born in 1909-1913 and then leveled off and slightly decreased in younger generations.

## 4. Discussion

Cervical cancer has been a major global burden that continues to increase largely due to aging, genetic factors, personal behavior, and other factors [14]. Furthermore, cervical cancer is preventable which is often caused by human papillomavirus (HPV) infection, particularly for two most common strains of HPV—16 and 18 [21]. HPV infection can result in precancerous lesions which increase the risk of cervical cancer [22]. Although the widespread implementation of the Papanicolaou (Pap) cytology test since the 1960s has reduced cervical cancer incidence and mortality in Canada and many high-income countries, the reductions have recently leveled off and disparities continue [23]. Thus, secular trends in cervical cancer mortality in the three selected high-income countries are important to assess the effect of public health measures such as cytology-based screening, HPV-based screening with Pap testing, and HPV vaccination [23].

Joinpoint analyses of cervical cancer mortality trends have already been successfully performed in many studies including Wang et al. [3], Li et al. [24], and Lee et al. [18]. Furthermore, the APC analyses of incidence and mortality trends for cervical cancer have also been reported for many areas such as Korea [25], Japan [26], and the United States [14]. However, a systematic comparison of these trends in Canada, Korea, and Italy using the same analytical models had been lacking. Thus, we conducted this study which focused on the comparison of cervical cancer mortality trends in Canada, Korea, and Italy using APC analysis methods to explore the cause of the cancer trends and assess the effect of public health control policies.

The overall trends observed in the three areas analyzed in our study suggested that the ASMR for cervical cancer in Canada and Italy decreased significantly during the period from 1986 to 2015, but leveled off thereafter. However, the trends for Korea showed a significant increase beginning in 1986 and a significant decrease after 2003. The significant decrease may have been caused by introduction of HPV vaccination into clinical practice in Korea in 2007, which resulted in a decreasing trend in the incidence of cervical cancer [27]. Moreover, the reductions in Canada and Italy were likely predominately related to the widespread implementation of Pap cytology testing during the mid-1950s and introduction of the HPV vaccine [14].

Our study revealed that the overall cervical cancer mortality rates in three countries generally increased with age and decreased with birth cohort during the whole observation period. The age effect on mortality rates of cervical cancer was an increase with advancing age for all three countries. The age effects revealed in the APC analysis for Korea and Italy generally increased significantly in groups younger than 50 years old and peaked in the age group of 50-54 years old; the age effect in Korea began to decrease after 55 years old except for a slight increase in the age group from 65-69 to 75-79 years old. This phenomenon indicated that cervical cancer mortality is trending more toward younger women in Korea and Italy [3]. The significant increasing rate observed before 50 years old indicated that the peak risk of incidence shifted to the middle-aged and younger groups, which was consistent with the findings of Li et al. [24]. Furthermore, a similar phenomenon was also observed in other medium-income countries, including China, as reported by Wei et al. [28]. The phenomenon might reveal that the cervical cancer mortality rate is rising at an alarming rate in younger women, and this phenomenon has become a global trend. Furthermore, the age effect for Canada was found to increase exponentially until age 55, when it continued to rise albeit at a slower pace. This indicated that older age has a higher mortality risk. Moreover, the coefficient of age effect for cancer mortality estimation became positive (greater than 0) starting at 40 years old. This further supports the fact that age has become a contributing factor for cervical cancer death in women over 40 years old [2]. After controlling for the period and cohort effects, the increasing age effect for older women may be explained by the high proportion of older adults in Canada [29].

In countries where organized cytological screening has been established over the past 40 years, cervical cancer incidence and mortality rates have declined steadily for many Western countries including the United States and Canada [21, 24]. Based on the APC analysis results in our study, period effects for Italy suggested that this screening had no beneficial effects on cervical cancer mortality. According to the results of the period effect on cervical cancer, there were net increases of 1.20 and 0.33 during the periods from 1986 to 2015 for Korea and Italy mortality rates, respectively. This might indicate that the period effect increased the risk of cervical cancer mortality for Korea and it might be an important factor affecting the trend of cervical cancer mortality for Korea [2, 30]. Moreover, although the Korean screening program was implemented in 1996, not all age groups received free access to the program at the same time. Thus, a decreased effect was observed after 2003 for Korea, which was likely predominately related to the widespread implementation of Pap cytology testing in 1996 [25]. Generally, such rapid increases might suggest that the screening program and early detection technology were not successful for some countries in Europe. The expected decreasing period effect might be affected by environmental deterioration and strong cohort effects such as HPV infection, HSV-2 infection, number of sexual partners, and younger age at first sexual intercourse for Korea and Italy [2, 31]. Contrarily, we found a decreased period effect in Canada, which can be explained by the combined effects of the promotion and implementation of cytology-based screening and HPV-based screening with Pap testing and implementation of the quadrivalent HPV vaccine in July 2006 for Canada [23].

On the whole, the risk by birth cohort in our study declined steadily among all birth cohorts for all three countries, except some birth cohorts that exhibited slightly increased risk during the observation period. Moreover, the effect of cohort for cervical cancer mortality rate peaked in the cohort born in 1909-1913 except in Korea, where it peaked in the birth cohort born from 1924 to 1928. For Canada and Italy, the risk of mortality began to decline in younger generations, suggesting a decreased risk of cervical cancer in younger generations. The decreasing cohort effects observed in younger generations might be explained by the decrease in the prevalence of cancer risk factors related to improvements in public health policy and socioeconomic status, access to hospital-based treatment, improvement of medical conditions, and implementation of cervical cancer screening programs that have occurred in recent years [14, 25]. However, a slight increase of cohort effect was still observed for both Italy and Canada in younger generations. This might be explained by the fact that women born in the 1950s and 1970s had altered sexual behavior compared to older women such as earlier age at first intercourse and number of sexual partners due to open sexuality and growth of sexual freedom, which may have increased the prevalence of HPV [31]. Additionally, young women in Italy and Canada might have a relatively lower screening rate for cervical cancer, and this rate presented a slight decreasing trend among younger generations [32]. Furthermore, the observed cohort effect for Korea was similar to that reported by Moon et al. [25]. Remarkably, the increased cohort effects were also observed in our study in older generations. This might be explained by the fact that older generations of Koreans had lower socioeconomic status in the 1910s to 1290s, and lower socioeconomic status was highly associated with risk of HPV infection, which is a major risk factor for cervical cancer [25]. Furthermore, the increased trend among younger cohorts might be explained by the fact that sexual behavior in younger cohorts has changed compared with that in older cohorts, and the age at first intercourse has become earlier in Korea [25]. Additionally, there have been some changes in the nature of cervical cancer in young women such as the increase of in situ adenocarcinoma or adenocarcinoma among young women from 1993 to 2001 in Korea, which led to relative hard detection in cervical cancer screening compared to squamous cell carcinoma [33].

Taken together, our study with joinpoint analysis revealed that the mortality rate of cervical cancer for Korea generally increased from 1986 to 2015, while the mortality rate generally decreased for Canada during the whole observation periods. The phenomenon might be explained by the fact that the clinical utilization rate of radiation therapy (RT) in Korea seems to be lower than that of the estimated optimal utilization rate of developed or developing countries [34]. Furthermore, the mortality rate for Italy exhibited a slow decline from 1988 and a slight increase beginning in 2005 through 2015. High mortality was always associated with high morbidity, thus, the decreased trend of cervical cancer mortality for Canada was probably due to effective treatment of early cervical cancer cases and the strong period and cohort effect due to improvements in public health policy and treatment of medical conditions in recent cohort years. However, the increased trend of cervical cancer mortality for Korea was probably due to the strong period effect and increased cohort effect in recent generations. Thus, the APC model in our study might reveal that the decrease in the cohort effect contributed to the reduced cancer mortality rate for Italy and Canada, and the increase of period effect and cohort effect in younger generations contributed to the increase in cancer mortality rate for Korea.

This study has some specific limitations. A major limitation is the lack of the comparison of incidence and mortality rate of cervical cancer in three high-income areas, as the prevention and control policies for cervical cancer are tailored to the local conditions. Thus, further analysis by APC analysis for cervical cancer incidence and mortality is needed in the future. Another limitation is the finite data of cervical cancer mortality from the WHO Mortality Database, leading to the relatively short study periods. This may have also resulted in imprecise estimations of the age-period-cohort effect on cervical cancer. Therefore, all results from the APC analysis in this study still need further confirmation with more incidence and mortality data.

## 5. Conclusions

In summary, we explored and assessed the secular trends of cervical cancer mortality rates for all female populations in Canada, Korea, and Italy during the periods from 1986 to 2015. Overall, declining trends were observed in the three countries before 2003, while the rates for Korea exhibited a significant increase during the periods from 1986 to 2003. Furthermore, the ASMR for Italy was the lowest during the whole observation periods, whereas the ASMR observed in Korea was higher than that of the other two countries beginning in 1994. Moreover, our APC analysis showed that the risk of cervical cancer mortality increased with age in all three countries, which indicated that advanced age might be a risk factor. In addition, our study revealed that the overall risks for mortality increased with period effects for Italy and Korea and generally decreased with birth cohort for the three areas. Taken together, these results show that the decrease in the cohort effect may have contributed to the reduced mortality, and the increase of period effect may have led to increased cancer mortality for Korea.

## Figures and Tables

**Table 1:** Joinpoint regression analysis of cervical cancer mortality in three high-income areas, 1986-2015.

Mortality	ASMR		AAPC (%)	Segment 1 APC (%)	Segment 2 APC (%)	Segment 3 APC (%)
	1986	2015				
Canada (1/100,000)	2.54	1.32	-1.9^∗^ (-2.4~-1.4)	1986-2008 -2.7^∗^ (-3.0~-2.4)	2008-2015 0.6 (-1.2~2.5)	
Korea (1/100,000)	1.03	2.00	2.2^∗^ (0.9~3.5)	1986-1989 18.9^∗^ (6.5~-32.7)	1989-2003 4.8^∗^ (3.6~6.0)	2003-2015 -4.5^∗^ (-5.7~-3.3)
Italy (1/100,000)	0.68	0.71	0.0 (-1.2~1.3)	1986-1988 16.4 (-2.1~38.5)	1988-2005 -2.8^∗^ (-3.5~-2.2)	2005-2015 1.9^∗^ (0.6~3.3)

APC: annual percent change; 95% CI: 95% confidence interval; AAPC: average annual percent change; ^∗^the APC and AAPC are significantly different from zero at alpha = 0.05 (*P* < 0.05).

**Table 2:** Results of APC model analysis for cervical cancer mortality in three areas.

Factor	Canada		Korea		Italy	
	Coef.	SE	Coef.	SE	Coef.	SE
Age (year)						
20-24	-2.51	1.00	-3.61	1.94	-3.18	2.42
25-29	-1.13	0.50	-1.67	0.77	-1.74	1.15
30-34	-0.45	0.39	-0.52	0.54	-0.57	0.76
35-39	-0.02	0.33	0.15	0.45	0.06	0.63
40-44	0.26	0.29	0.48	0.39	0.44	0.53
45-49	0.48	0.26	0.69	0.33	0.63	0.47
50-54	0.46	0.24	0.74	0.28	0.78	0.40
55-59	0.53	0.22	0.67	0.24	0.75	0.36
60-64	0.55	0.20	0.65	0.20	0.66	0.33
65-69	0.56	0.19	0.62	0.18	0.68	0.31
70-74	0.64	0.19	0.78	0.18	0.73	0.32
75-79	0.63	0.22	1.03	0.20	0.75	0.36
Period (year)						
1986-1990	0.10	0.16	-0.76	0.21	-0.11	0.29
1991-1995	0.03	0.14	-0.28	0.15	-0.04	0.23
1996-2000	0.05	0.14	0.04	0.12	-0.01	0.21
2001-2005	-0.07	0.15	0.44	0.11	-0.06	0.23
2006-2010	-0.10	0.16	0.32	0.14	0.01	0.26
2011-2015	-0.01	0.17	0.24	0.18	0.22	0.27
Cohort (year)						
1909-1913	0.77	0.38	0.08	0.59	1.01	0.65
1914-1918	0.68	0.29	0.40	0.43	0.88	0.53
1919-1923	0.62	0.25	0.68	0.36	0.72	0.47
1924-1928	0.34	0.25	0.97	0.32	0.50	0.44
1929-1933	0.21	0.24	0.86	0.31	0.33	0.43
1934-1938	0.10	0.24	0.67	0.32	0.11	0.44
1939-1943	0.04	0.27	0.51	0.35	-0.01	0.48
1944-1948	-0.09	0.30	0.16	0.39	-0.07	0.53
1949-1953	-0.12	0.32	-0.10	0.43	-0.18	0.59
1954-1958	-0.10	0.35	-0.24	0.47	-0.15	0.64
1959-1963	-0.20	0.38	-0.36	0.51	-0.30	0.71
1964-1968	-0.24	0.42	-0.66	0.58	-0.47	0.81
1969-1973	-0.23	0.47	-0.76	0.65	-0.37	0.89
1974-1978	-0.34	0.57	-0.70	0.73	-0.55	1.09
1979-1983	-0.47	0.76	-0.47	0.87	-0.31	1.31
1984-1988	-0.51	1.14	-0.31	1.36	-0.54	2.34
1989-1993	-0.43	2.74	-0.72	5.04	-0.60	6.07
Deviance	0.99		2.36		0.40	
AIC	3.78		3.90		3.01	
BIC	-170.27		-168.71		-170.66	

## Data Availability

The mortality data used to support the findings of our study were available at WHO Mortality Database in three countries including Canada, Korea, and Italy (https://www-dep.iarc.fr/WHOdb/WHOdb.htm). This database contains selected cancer mortality statistics by country, extracted from the World Health Organisation (WHO) database. And all data here were open for public. The original data have been converted and/or recoded to a common system before presentation. In our study, the age-specific mortality rate data for cervical cancer by year in Canada, Korea, and Italy are listed as Tables S1–S3 in Supplementary Materials.

## Supplementary Materials

Supplementary Materials Table S1: age-specific incidence rates (per 100,000 women) of cervical cancer in Canada. Table S2: age-specific mortality rates (per 100,000 women) of cervical cancer in Korea. Table S3: age-specific mortality rates (per 100,000 women) of cervical cancer in Italy.

## References

[1] Foley G., Alston R., Geraci M., Brabin L., Kitchener H., Birch J. (2011). Increasing rates of cervical cancer in young women in England: an analysis of national data 1982–2006. *British Journal of Cancer*.

[2] Wang J., Lv H., Xue Z., Wang L., Bai Z. (2018). Temporal trends of common female malignances on breast, cervical, and ovarian cancer mortality in Japan, Republic of Korea, and Singapore: application of the age-period-cohort model. *BioMed Research International*.

[3] Wang T., Wu M. H., Wu Y. M., Zhang W. Y. (2015). A population-based study of invasive cervical cancer patients in Beijing. *Chinese Medical Journal*.

[4] Torre L. A., Bray F., Siegel R. L. (2015). Global cancer statistics, 2012. *CA: a Cancer Journal for Clinicians*.

[5] Globocan cancer fact sheet. https://gco.iarc.fr/today/fact-sheets-cancers.

[6] Chen W., Zheng R., Zeng H., Zhang S. (2015). The updated incidences and mortalities of major cancers in China, 2011. *Chinese Journal of Cancer*.

[7] Wojtyla C., Janik-Koncewicz K., La Vecchia C. (2020). Cervical cancer mortality in young adult European women. *European Journal of Cancer*.

[8] Levin C. E., Sharma M., Olson Z. (2015). An extended cost-effectiveness analysis of publicly financed HPV vaccination to prevent cervical cancer in China. *Vaccine*.

[9] Canfell K., Shi J. F., Lew J. B. (2011). Prevention of cervical cancer in rural China: evaluation of HPV vaccination and primary HPV screening strategies. *Vaccine*.

[10] Teixeira C., Afonso A., Rodrigues L., Madureira M., Nogueira A. (2019). Incidence and mortality due to cervical cancer in 4 south European countries. *Porto Biomedical Journal*.

[11] Bosetti C., Bertuccio P., Levi F., Lucchini F., Negri E., la Vecchia C. (2008). Cancer mortality in the European Union, 1970-2003, with a joinpoint analysis. *Annals of Oncology*.

[12] Kim H. J., Fay M. P., Feuer E. J., Midthune D. N. (2000). Permutation tests for joinpoint regression with applications to cancer rates. *Statistics in Medicine*.

[13] Yang Y., Fu W. J., Land K. C. (2016). 2. A methodological comparison of age-period-cohort models: the intrinsic estimator and conventional generalized linear Models. *Sociological Methodology*.

[14] Wang J., Bai Z., Wang Z., Yu C. (2016). Comparison of secular trends in cervical cancer mortality in China and the United States: an age-period-cohort analysis. *International Journal of Environmental Research and Public Health*.

[15] Wang Z., Bao J., Yu C., Wang J., Li C. (2015). Secular trends of breast cancer in China, South Korea, Japan and the United States: application of the age-period-cohort analysis. *International Journal of Environmental Research and Public Health*.

[16] Segi M. (1960). Cancer mortality for selected sites in 24 countries[R]. *Department of Public Health*.

[17] Doll R., Payne P. M., Waterhouse J. A. H. (1966). *Cancer Incidence in Five Continents[R]*.

[18] Lee J. Y., Kim E. Y., Jung K. W. (2014). Trends in gynecologic cancer mortality in east Asian regions. *Journal of Gynecologic Oncology*.

[19] Fu W. J. (2000). Ridge estimator in singulah oesiun with application to age-period-cohort analysis of disease rates. *Communications in Statistics—Theory and Method*.

[20] Yang Y., Schulhofer-Wohl S., Fu W. J., Land K. C. (2008). The intrinsic estimator for age-period-cohort analysis: what it is and how to use it. *American Journal of Sociology*.

[21] Park Y., Vongdala C., Kim J., Ki M. (2015). Changing trends in the incidence (1999-2011) and mortality (1983-2013) of cervical cancer in the Republic of Korea. *Epidemiol Health*.

[22] Kau Y. C., Liu F. C., Kuo C. F. (2019). Trend and survival outcome in Taiwan cervical cancer patients. *Medicine (Baltimore)*.

[23] Saraiya M., Steben M., Watson M., Markowitz L. (2013). Evolution of cervical cancer screening and prevention in United States and Canada: implications for public health practitioners and clinicians. *Preventive Medicine*.

[24] Li X., Zheng R., Li X. (2017). Trends of incidence rate and age at diagnosis for cervical cancer in China, from 2000 to 2014. *Chinese Journal of Cancer Research*.

[25] Moon E. K., Oh C. M., Won Y. J. (2017). Trends and age-period-cohort effects on the incidence and mortality rate of cervical cancer in Korea. *Cancer Research and Treatment*.

[26] UCHIDA H., KOBAYASHI M., HOSOBUCHI A. (2014). Age, period, and birth cohort-specific effects on cervical cancer mortality rates in Japanese women and projections for mortality rates over 20-year period (2012–2031). *Nihon Eiseigaku Zasshi*.

[27] Lim M. C., Moon E. K., Shin A. (2013). Incidence of cervical, endometrial, and ovarian cancer in Korea, 1999-2010. *Journal of Gynecologic Oncology*.

[28] Wei M., Zhou W., Bi Y., Wang H., Liu Y., Zhang Z. J. (2019). Rising mortality rate of cervical cancer in younger women in urban China. *Journal of General Internal Medicine*.

[29] Luo L., Jiang J., Zhang G. (2017). Stroke mortality attributable to ambient particulate matter pollution from 1990 to 2015 in China: an age-period-cohort and spatial autocorrelation analysis. *International Journal of Environmental Research and Public Health*.

[30] Li C., Yu C., Wang P. (2015). An age-period-cohort analysis of female breast cancer mortality from 1990-2009 in China. *International Journal for Equity in Health*.

[31] Vaccarella S., Franceschi S., Engholm G., Lönnberg S., Khan S., Bray F. (2014). 50 years of screening in the Nordic countries: quantifying the effects on cervical cancer incidence. *British Journal of Cancer*.

[32] Suh M., Choi K. S., Lee Y. Y., Jun J. K. (2013). Trends in cancer screening rates among Korean men and women: results from the Korean National Cancer Screening Survey, 2004-2012. *Cancer Research and Treatment*.

[33] The International Collaboration of Epidemiological Studies of Cervical Cancer (2007). Comparison of risk factors for invasive squamous cell carcinoma and adenocarcinoma of the cervix: collaborative reanalysis of individual data on 8,097 women with squamous cell carcinoma and 1,374 women with adenocarcinoma from 12 epidemiological studies. *International Journal of Cancer*.

[34] Seo Y. S., Kim M. S., Kang J. K. (2018). The clinical utilization of radiation therapy in Korea between 2011 and 2015. *Cancer Research and Treatment*.

