# Supplementary material for: The Effects of Age, Period, and Cohort on the Mortality of Cervical Cancer in Three High-Income Countries: Canada, Korea, and Italy
Source: Biomed Res Int. 2021 Jan 4;2021:8829122. doi: 10.1155/2021/8829122 (PMC7803425; doi:10.1155/2021/8829122)
Supplement: Supplementary Materials — Table S1: age-specific incidence rates (per 100,000 women) of cervical cancer in Canada. Table S2: age-specific mortality rates (per 100,000 women) of cervical cancer in Korea. Table S3: age-specific mortality rates (per 100,000 women) of cervical cancer in Italy. [file 8829122.f1.docx]

**Table S1.** Age-specific incidence rates (per 100, 000 women) of cervical cancer in Canada

| **Age** | **Year of onset** | | | | | |
| --- | --- | --- | --- | --- | --- | --- |
|  | 1986-1990 | 1991-1995 | 1996-2000 | 2001-2005 | 2006-2010 | 2011-2015 |
| 20-24 | 0.22 | 0.10 | 0.18 | 0.17 | 0.07 | 0.13 |
| 25-29 | 0.81 | 0.71 | 0.64 | 0.60 | 0.37 | 0.54 |
| 30-34 | 1.71 | 1.44 | 1.19 | 1.18 | 1.10 | 1.07 |
| 35-39 | 2.32 | 2.50 | 2.38 | 1.80 | 1.78 | 1.70 |
| 40-44 | 3.28 | 2.99 | 3.22 | 2.55 | 2.27 | 2.85 |
| 45-49 | 4.49 | 4.42 | 3.67 | 3.44 | 2.91 | 3.53 |
| 50-54 | 5.46 | 3.76 | 3.62 | 3.85 | 3.53 | 3.22 |
| 55-59 | 5.55 | 5.19 | 5.00 | 4.06 | 3.52 | 3.53 |
| 60-64 | 6.99 | 6.05 | 5.13 | 4.13 | 3.75 | 3.88 |
| 65-69 | 9.63 | 6.49 | 5.94 | 4.45 | 4.57 | 3.61 |
| 70-74 | 10.72 | 8.89 | 7.24 | 5.51 | 4.69 | 5.74 |
| 75-79 | 11.53 | 9.70 | 9.51 | 6.29 | 5.66 | 5.05 |

**Table S2.** Age-specific mortality rates (per 100, 000 women) of cervical cancer in Korea

| **Age** | **Year of onset** | | | | | |
| --- | --- | --- | --- | --- | --- | --- |
|  | 1986-1990 | 1991-1995 | 1996-2000 | 2001-2005 | 2006-2010 | 2011-2015 |
| 20-24 | 0.04 | 0.03 | 0.01 | 0.05 | 0.07 | 0.04 |
| 25-29 | 0.15 | 0.25 | 0.25 | 0.29 | 0.34 | 0.42 |
| 30-34 | 0.59 | 1.00 | 0.72 | 0.75 | 0.93 | 1.18 |
| 35-39 | 1.36 | 1.83 | 2.19 | 1.76 | 1.59 | 1.89 |
| 40-44 | 2.14 | 2.71 | 3.50 | 3.37 | 2.75 | 2.73 |
| 45-49 | 3.68 | 4.77 | 4.82 | 4.86 | 4.33 | 3.55 |
| 50-54 | 4.84 | 5.72 | 6.45 | 6.36 | 5.61 | 5.09 |
| 55-59 | 5.09 | 6.54 | 8.73 | 8.40 | 5.74 | 4.91 |
| 60-64 | 5.72 | 7.93 | 9.90 | 11.64 | 6.95 | 5.69 |
| 65-69 | 3.67 | 8.71 | 10.61 | 14.25 | 10.98 | 6.34 |
| 70-74 | 3.75 | 8.29 | 12.53 | 20.25 | 14.71 | 10.59 |
| 75-79 | 3.42 | 7.51 | 13.68 | 29.33 | 21.51 | 15.12 |

**Table S3.** Age-specific mortality rates (per 100, 000 women) of cervical cancer in Italy

| **Age** | **Year of onset** | | | | | |
| --- | --- | --- | --- | --- | --- | --- |
|  | 1986-1990 | 1991-1995 | 1996-2000 | 2001-2005 | 2006-2010 | 2011-2015 |
| 20-24 | 0.01 | 0.02 | 0.01 | 0.06 | 0.03 | 0.03 |
| 25-29 | 0.10 | 0.13 | 0.16 | 0.06 | 0.12 | 0.12 |
| 30-34 | 0.52 | 0.40 | 0.37 | 0.30 | 0.30 | 0.49 |
| 35-39 | 0.81 | 0.88 | 0.78 | 0.55 | 0.70 | 0.83 |
| 40-44 | 1.27 | 1.40 | 1.25 | 0.95 | 0.94 | 1.39 |
| 45-49 | 1.60 | 1.76 | 1.45 | 1.46 | 1.46 | 1.50 |
| 50-54 | 2.12 | 1.90 | 1.99 | 1.75 | 2.01 | 2.07 |
| 55-59 | 2.51 | 2.42 | 1.96 | 1.98 | 1.71 | 2.16 |
| 60-64 | 2.72 | 2.53 | 2.14 | 1.96 | 1.83 | 1.93 |
| 65-69 | 3.87 | 3.13 | 2.81 | 1.95 | 1.84 | 2.07 |
| 70-74 | 4.42 | 3.75 | 3.37 | 2.73 | 2.41 | 2.78 |
| 75-79 | 5.11 | 4.88 | 4.30 | 3.28 | 2.90 | 2.80 |
